# Enfermedad relacionada con IgG4: a propósito de un caso

**DOI:** 10.1515/almed-2019-0002

**Published:** 2020-01-07

**Authors:** Silvia de las Heras Flórez, Mercedes Carretero Pérez, Carmen Teresa Sanz Díaz, José Alejandro Medina García

**Affiliations:** Unidad Core-Proteínas, Hospital Universitario Nuestra Señora de la Candelaria, Carretera General del Rosario 145, 38010 Santa Cruz de Tenerife, Spain; Medicina Interna, Hospital Universitario Nuestra Señora de Candelaria, Santa Cruz de Tenerife, Spain

**Keywords:** infiltrado linfoplasmocítico, inmunoglobulina IgG, inmunoglobulina IgG4

## Abstract

La enfermedad relacionada con IgG4 (ER-IgG4) es un proceso patológico de reciente descripción que puede afectar a casi cualquier órgano. Su diagnóstico se basa en la correlación de hallazgos clínicos con los resultados histopatológicos, así como en el hallazgo de la IgG4 sérica elevada. El tratamiento se basa en corticoides y rituximab en los casos más graves. Presentamos el caso de un paciente con un cuadro sistémico, diagnosticado de ER-IgG4 en el que el hallazgo de niveles elevados de IgG4 en suero sirvieron como apoyo en el diagnóstico.

## Introducción

La enfermedad relacionada con la IgG4 (ER-IgG4) es una entidad clínico-patológica de reciente descripción que puede presentar un amplio espectro de manifestaciones clínicas. El cuadro clínico se caracteriza por la presencia de lesiones tumefactas, un infiltrado denso linfoplasmacítico con abundantes células plasmáticas positivas para IgG4, fibrosis estoriforme, flebitis obliterativa y frecuentemente, concentraciones séricas de IgG4 elevadas [1]. La prevalencia e incidencia de la enfermedad son poco conocidas, y probablemente estén siendo subestimadas.

La primera descripción de esta entidad se realizó en el año 2001 cuando se relacionó en un paciente con pancreatitis autoinmune, la elevación de IgG4 sérica con la presencia de numerosas células linfoplasmocíticas IgG4 [2]. En el año 2003 se definió como una nueva entidad clínico patológica con afectación sistémica [3] y en 2012 se propuso el término enfermedad relacionada con IgG4 que se emplea actualmente [4]. Dicha entidad es un proceso patológico que ha unificado un gran número de enfermedades consideradas como propias.

El diagnóstico se realiza generalmente entre la sexta y séptima décadas de la vida, y es más frecuente en los varones. El órgano más afectado es el páncreas aunque puede afectarse cualquier otra parte anatómica [5], [6], [7], [8], como las glándulas salivales, riñón, aorta, pulmón y retroperitoneo.

La presentación es subaguda y los síntomas constitucionales son infrecuentes, predominando los síntomas derivados de la alteración del órgano afectado. Son frecuentes los antecedentes de rinitis, asma, pólipos nasales y atopia [9].

El diagnóstico puede ser difícil, tanto por presentar una sintomatología inespecífica, como por sugerir otras enfermedades más comunes. La sintomatología y los hallazgos de laboratorio orientan al médico clínico para dirigir las exploraciones complementarias y poder llegar al diagnóstico.

## Caso clínico

El paciente es un varón de 29 años con antecedentes de rinitis alérgica, que consulta a su médico de Atención Primaria en diciembre del año 2015 por presentar una astenia de 6 meses de evolución y una pérdida de peso de 10 kilos.

La exploración física fue anodina y tras realizar las pruebas de laboratorio se observaron las siguientes alteraciones bioquímicas sanguíneas: elevación de la concentración de creatinina (1.95 mg/dL; rango de referencia: 0.70–1.20 mg/dL), eosinofilia (3.51 ×10^3^ células/μL; rango de referencia: <0.50 células/μL), elevación de las enzimas de colestasis gamma-glutamil transferasa (263 U/L; rango de referencia: 11–50 U/L) y fosfatasa alcalina (523 UI/L; rango de referencia: 40–129 UI/L), así como una concentración de proteína de 9.2 g/dL (rango de referencia: 6.6–8.7 g/dL). Aunque no se solicitaba la medición de la bilirrubina, el índice de ictericia era inferior al límite de referencia.

Los datos analíticos y la sintomatología clínica que presentaba el paciente motivaron su derivación a las consultas externas de Medicina Interna para su estudio. En la consulta de Medicina Interna, se planteó realizar el diagnóstico diferencial entre vasculitis, proceso linfoproliferativo y proceso parasitario.

En la exploración analítica se observó una marcada elevación de la concentración de IgG (4.132 mg/dL; rango de referencia: 700–1.600 mg/dL) y de IgE (1.281 UI/mL; rango de referencia: <100 UI/mL). El resto del estudio analítico era normal, incluidos los marcadores de autoinmunidad (anticuerpos antinucleares, anticuerpos anti antígenos nucleares extraíbles y anticuerpos anticitoplasma de neutrófilo), serologías (ameba, Strongyloide, Trypanosoma Cruzi), CA19-9 (3.6 UI/mL; rango de referencia: <37 UI/mL), adenosina desaminasa, crioglobulinas, complemento y prueba de Mantoux.

En el estudio radiológico se solicitó una tomografía computarizada toraco-abdomino-pélvica, que evidenció el hallazgo de múltiples adenopatías de tamaño significativo e infiltración linfomatosa renal y periportal, orientando como primera posibilidad diagnóstica hacia un proceso linfoproliferativo.

Se solicitó una interconsulta con los servicios de Nefrología y de Cirugía General para realizar una biopsia renal y una exéresis quirúrgica de un ganglio inguinal para realizar el estudio anatomopatológico. Los resultados de ambos estudios descartaban la existencia de un proceso linfoproliferativo, aunque se describía la presencia de un importante incremento de la celularidad linfoplasmocitaria. Para realizar una ampliación del estudio, las muestras fueron remitidas al centro de referencia, donde después de realizar los estudios inmunohistoquímicos complementarios, se recomendó que se descartara la existencia de una enfermedad relacionada con la IgG4.

El estudio de las subclases de la IgG informaba de una concentración de IgG total de 2.225 mg/dL, IgG1 de 1.432 mg/dL (rango de referencia: 382–929 mg/dL); IgG2 de 816 mg/dL (rango de referencia: 242–700 mg/dL); IgG3 de 184 mg/dL (rango de referencia: 22–176 mg/dL) e IgG4: 652 mg/dL (rango de referencia: 4–86 mg/dL).

El diagnóstico final se obtuvo al descartar otras entidades patológicas diferentes de la ER-IgG4, así como también por los resultados de las biopsias compatibles y la observación de una IgG4 muy elevada.

Durante el tiempo del estudio, al paciente se le administraron 3 bolos de 125 mg de metilprednisolona, así como prednisona por vía oral (10 mg/día). Al establecer el diagnóstico de ER-IgG4, se decidió iniciar la terapia con rituximab, comenzando en noviembre del año 2016 (2 ciclos de 1 gramo cada 6 meses); la razón fue que los corticoides producían una mejoría clínica parcial, presentando además una reactivación del herpes zoster, hipertensión arterial y hábito cushingoide. En mayo del año 2017 se administran los siguientes 2 ciclos.

El paciente experimentó una mejoría clínica progresiva, con normalización de las cifras de creatinina y valores de IgG4 inferiores a 135 mg/dL tras los 4 primeros ciclos de rituximab, así como también se observó una disminución significativa del tamaño de las adenopatías y del infiltrado en la pruebas de imagen.

## Discusión

La ER-IgG4 es una entidad de reciente descripción cuyo diagnóstico puede ser difícil, siendo una entidad cada vez más planteada en el diagnóstico diferencial de una gran variedad de cuadros clínicos que cursan con afectación de un sólo órgano o sistémica [10], [11].

La etiopatogenia de esta enfermedad permanece poco clara, incluido el papel que tiene la IgG4, aunque la hipótesis más aceptada es que está inducida por fenómenos de autoinmunidad [12].

A pesar de que el diagnóstico definitivo se realiza mediante la biopsia del órgano afectado y su estudio inmunohistoquímico, el papel del laboratorio en el diagnóstico de la ER-IgG4 es importante, ya que existen criterios diagnósticos propuestos por varios grupos, no validados, en los que el hallazgo de unos valores de IgG4 mayores de 135 mg/dL se consideran como altamente sugestivos de esta entidad [6], [12], [13].

Los niveles séricos aumentados de IgG4 para el diagnóstico de esta enfermedad se considera que tienen una sensibilidad y valor predictivo positivo altos (>90%), pero con una especificidad y valor predictivo negativo insuficientes [14]. La limitación de la medición de IgG4 sérica en esta entidad, es que hay casos en los que puede no estar elevada, y que puede estar elevada en otras enfermedades tales como bronquiectasias, asma, sarcoidosis y adenocarcinoma de páncreas, entre otras.

Otra función de la medición de las concentraciones de IgG4 en suero es la de monitorizar la respuesta del paciente al tratamiento, aunque los datos en las publicaciones científicas son discordantes sobre esta utilidad [15], [16]. En el caso clínico descrito, los valores fueron concordantes con la evolución clínica y la respuesta al tratamiento, aunque actualmente no existe suficiente evidencia científica para justificar este uso.

Existen muchas incógnitas sin aclarar respecto a la ER-IgG4, tanto sobre su etiopatogenia como sobre el papel de la IgG4 en esta entidad. La medición de los niveles séricos de IgG4 en el laboratorio, no tiene suficiente evidencia para sacar conclusiones respecto a su papel en el diagnóstico, aunque su uso en un contexto clínico adecuado, y especialmente si su concentración es el doble del límite de referencia, puede ser de utilidad para dirigir los estudios complementarios y llegar al diagnóstico. Se necesitan más evidencias para recomendar su uso como marcador pronóstico y de respuesta al tratamiento.

Aunque se están estudiando otros biomarcadores que podrían ser utilizados en un futuro para diagnosticar esta entidad, son necesarios más estudios y evidencias [17].

A pesar de que el paciente era joven y su clínica era inespecífica, los hallazgos histopatológicos y analíticos fueron determinantes para llegar al diagnóstico de ER-IgG4 en apenas 6 meses de evolución de su cuadro clínico, teniendo en cuenta que la media de retraso del diagnóstico puede ser de 4–5 años, especialmente por el gran papel de simuladora que tiene esta entidad cuyo diagnóstico es creciente.
